# Contrasting defense strategies of oligotrophs and copiotrophs revealed by single-cell-resolved virus–host pairing of freshwater bacteria

**DOI:** 10.1093/ismeco/ycaf086

**Published:** 2025-05-21

**Authors:** Yusuke Okazaki, Yohei Nishikawa, Ryota Wagatsuma, Haruko Takeyama, Shin-ichi Nakano

**Affiliations:** Institute for Chemical Research, Kyoto University, Gokasho, Uji, Kyoto 611-0011, Japan; Computational Bio Big-Data Open Innovation Laboratory, AIST-Waseda University, 3-4-1 Okubo, Shinjuku-ku, Tokyo 169-0072, Japan; Research Organization for Nano & Life Innovation, Waseda University, 513 Waseda Tsurumaki-cho, Shinjuku-ku, Tokyo 162-0041, Japan; Computational Bio Big-Data Open Innovation Laboratory, AIST-Waseda University, 3-4-1 Okubo, Shinjuku-ku, Tokyo 169-0072, Japan; Graduate School of Advanced Science and Engineering, Waseda University, 2-2 Wakamatsu-cho, Shinjuku-ku, Tokyo 162-8480, Japan; Computational Bio Big-Data Open Innovation Laboratory, AIST-Waseda University, 3-4-1 Okubo, Shinjuku-ku, Tokyo 169-0072, Japan; Research Organization for Nano & Life Innovation, Waseda University, 513 Waseda Tsurumaki-cho, Shinjuku-ku, Tokyo 162-0041, Japan; Graduate School of Advanced Science and Engineering, Waseda University, 2-2 Wakamatsu-cho, Shinjuku-ku, Tokyo 162-8480, Japan; Institute for Advanced Research of Biosystem Dynamics, Waseda Research Institute for Science and Engineering, Graduate School of Advanced Science and Engineering, Waseda University, 3-4-1 Okubo, Shinjuku-ku, Tokyo 169-8555, Japan; Center for Ecological Research, Kyoto University 2-509-3 Hirano, Otsu, Shiga 520-2113, Japan

**Keywords:** virus–host interaction, single-cell genomics, lake bacterioplankton

## Abstract

Characterizing virus–host pairs and the infection state of individual cells is the major technical challenge in microbial ecology. We addressed these challenges using state-of-the-art single-cell genome technology (SAG-gel) combined with extensive metagenomic datasets targeting the bacterial and viral communities in Lake Biwa. From two water layers and two seasons, we obtained 862 single-cell amplified genomes (SAGs), including 176 viral (double-stranded DNA phage) contigs, which identified novel virus–host pairs involving dominant freshwater lineages. The viral infection rate, estimated by mapping the individual SAG’s raw reads to viral contigs, showed little variation among samples (12.1%–18.1%) but significant variation in host taxonomy (4.2%–65.3%), with copiotrophs showing higher values than oligotrophs. The high infection rates of copiotrophs were attributed to collective infection by diverse viruses, suggesting weak density-dependent virus–host selection, presumably due to their nonpersistent interactions with viruses resulting from fluctuating abundance. In contrast, the low infection rates of oligotrophs supported the idea that their codominance with viruses is achieved by genomic microdiversification, which diversifies the virus–host specificity, sustained by their large population size and persistent density-dependent fluctuating selection. Notably, we discovered viruses infecting CL500-11, the dominant bacterioplankton lineage in deep freshwater lakes worldwide. These viruses showed extremely high read coverages in cellular and virion metagenomes but were detected in <1% of host cells, suggesting a low infection rate and high burst size. Overall, we revealed highly diverse virus–host interactions within and between host lineages that were overlooked at the metagenomic resolution.

## Introduction

Viruses are major mortality factors of bacteria [1] and primary drivers of genome diversification resulting from a complex virus–host arms race [2]. Although metagenomics allows the recovery of thousands of uncultured microbial and viral genomes from the environment, virus–host interactions remain unclear due to two technical limitations. The first limitation is the difficulty of host prediction. Much effort has focused on linking virus and host using metagenomic information, for example, through sequence matching, nucleotide frequency, similarity to isolated viruses, lineage-specific marker genes, and co-occurrence with potential hosts [3, 4]. Nucleotide sequence matching between viruses and hosts includes the detection of recombination sites, clustered regularly interspaced short palindromic repeat (CRISPR) spacers, conserved genetic regions, and integrated prophages, enabling sensitive prediction of virus–host pairs [4]. However, such strong evidence is rarely available, and the host remains unknown for the vast majority of metagenome-assembled viral genomes [5].

The second limitation is the difficulty in resolving the viral infection status of individual host cells. Even when the host is predicted from metagenomic information, it does not confirm the association of the virus and host at the time of sampling. This is particularly true when the host is predicted by a CRISPR spacer match, as it indicates that the host is immune to the virus due to a past infection event [3, 4]. Moreover, unlike experimental conditions, cells of a natural host population are not uniformly infected by viruses due to their physiological and genetic heterogeneity. However, metagenomics overlooks such intrapopulation heterogeneity because it targets populations of cells and virions. Therefore, the estimation of the viral infection rate of environmental bacteria generally requires experimental or microscopic approaches [6–8].

Several other techniques have been developed for studying environmental virus–host interactions. For instance, high-throughput chromosome conformation capture (Hi-C) combined with metagenomics chemically links virus and host chromosomes co-existing in a cell, detecting novel virus–host associations in various environments [9–11]. However, since this method also relies on metagenomics, it does not resolve the intrapopulation heterogeneity of virus–host interactions. Nonmetagenomic techniques such as fluorescence *in situ* hybridization [8, 12] and epicPCR [13, 14] allow direct investigation of virus–host associations in individual cells. However, these are targeted approaches using selected probes or marker genes, which limit their applicability to uncultured and diverse environmental viruses and hosts.

Single-cell genomics enables nontargeted, genome-resolved virus–host detection at single-cell resolution. By detecting viral signals in individual single-cell amplified genomes (SAGs), previous studies have predicted virus–host pairs and estimated their infection rates in marine [15–17] and hot spring [18, 19] systems. The major challenge in SAG-based approaches is the bias introduced by the whole genome amplification [20–23], which limits the number of qualified SAGs available for downstream analyses. For instance, the aforementioned single-cell viral detection studies analyzed at most 253 SAGs in a single study [19].

In the present study, leveraging state-of-the-art gel bead-based SAG technology (SAG-gel) [24], we investigated the interaction between bacterioplankton and their virus (double-stranded DNA phages) at single-cell resolution in a freshwater lake. In SAG-gel, individual cells are first encapsulated in a gel bead, where DNA extraction and whole-genome amplification of individual cells are performed within each bead. Only gel beads showing sufficient DNA amplification are sorted and sequenced, enabling efficient and high-quality reconstruction of SAGs with a reduced risk of contamination [24, 25]. Using this technique, we generated 862 SAGs available for downstream viral detection analyses.

We targeted the microbial ecosystem of Lake Biwa, Japan, where metagenomic studies have already reconstructed >500 bacterial and >7000 viral genomes and characterized their spatiotemporal distributions [26–29]. Although these studies identified quantitatively significant bacteria and viruses in the lake, extensive efforts to link viruses and hosts using metagenomic information resulted in limited success. For example, Actinobacteria and their viruses are the most abundant and diverse members of the microbial ecosystem in Lake Biwa and in freshwater lakes globally [26, 30, 31]. However, their virus–host specificity and infection rate in the ecosystem, which are key to understanding the mechanisms behind virus–host codominance and their sustainable diversity, remain unknown. Similarly, CL500-11, which is among the most abundant freshwater bacterioplankton lineages dominating in the oxygenated hypolimnion of deep freshwater lakes [32–35], has no known viruses, despite exhaustive metagenomic efforts to find them [26]. This is particularly intriguing given their potential ecological importance in controlling the seasonally fluctuating dynamics of the host population [32, 34].

Leveraging our 862 SAGs and published metagenomic data spatiotemporally collected from the lake [26–28], we performed an untargeted investigation of virus–host interactions at single-cell resolution. The results identified numerous novel virus–host pairs and demonstrated cell-to-cell heterogeneity in virus–host interactions. Comparative analyses among samples and host lineages highlighted the differences in viral infection rates between oligotrophic and copiotrophic hosts, suggesting that the persistence of virus-host interactions is key to determining viral infection dynamics and host defense strategies.

## Materials and methods

### Sample collection and single-cell genome sequencing

Microbial samples were collected near the deepest point of Lake Biwa, Japan (water depth, ~101 m) (35°20.10 N 136°06.16E), in summer (29 June 2022) and winter (1 February 2023). Vertical profiles of temperature and dissolved oxygen concentration (DO) were analyzed *in situ* using a conductivity, temperature, and depth (CTD) probe (Rinko ASTD102; JFE Advantech, Tokyo, Japan) to determine the sampling depths. In summer, the water was thermally stratified, with a surface water temperature of 26°C. In winter, the water column was almost holomictic, with an oxycline at ~85 m depth, where DO decreased from 10.1 to 3.9 mg l^−1^ (Fig. S1). In both seasons, we collected two samples representing the epilimnion and hypolimnion (5 and 80 m in summer and 5 and 95 m in winter). For each sample, 1 l of lake water was collected and kept under cool, dark conditions and processed within 24 h. Concentration, gel bead encapsulation, DNA extraction, whole-genome amplification, fluorescent staining, sorting, and sequencing of individual cells were performed following a published SAG-gel protocol [24]. For each sample, we sorted 192 and 768 gel beads in summer and winter, respectively, for a total of 1920 gel beads. For each gel bead, 0.6–546.6 Mb of reads (median = 46.3 Mb) were generated by Illumina NextSeq 2000 paired-end sequencing (2 × 150 bp). For each assembly, we labeled the samples “LB2206” for summer and “LB2301” for winter, and appended an E or H for epilimnion or hypolimnion samples, respectively, followed by a numerical identifier.

### Generation and taxonomic annotation of single-cell amplified genomes

The raw reads from each gel bead were preprocessed by bbduk v38.96, using the parameters ktrim = r ref = adapters k = 23 mink = 11 hdist = 1 tpe tbo qtrim = r trimq = 10 minlength = 40 maxns = 1 minavgquality = 15 (https://sourceforge.net/projects/bbmap/). Assembly was conducted using SPAdes v3.15.2, using the parameters --sc --careful --disable-rr [36]. Using SeqKit v2.4.0 [37], we removed <1 kb contigs and calculated the N50 of each assembly. The quality and taxonomy of the genomes were evaluated by CheckM v1.2.2 [38] and GTDB-Tk v2.2.6 (reference data version = r207) [39, 40], respectively. In the analysis, we replaced the Genome Taxonomy Database (GTDB) family name “UBA11657” with “CL500-11,” which is a familiar name for this uncultured bacterioplankton lineage [34, 41]. Based on the CheckM results, we defined a quality score (QS) for each assembly as “Completeness – 5 × Contamination,” which is a metric commonly used to evaluate the quality of metagenome-assembled genomes (MAGs) [40, 42]. SAGs with QS > 30 were used in the downstream analyses. The circularity of an assembly was tested using ccfind v1.4.5 [43]. A pangenome graph of SAGs was generated using SuperPang v1.1.0 [44] using the default parameters, and visualized by Bandage v0.9.0 [45].

### Identification of viral contigs in a single-cell amplified genomes

We used geNomad v1.5.1 [46], VIBRANT v1.2.1 [47], and VirSorter2 v2.2.3 [48] to identify double-stranded DNA (dsDNA) phage contigs in each SAG. In geNomad, we used the “end-to-end” pipeline with the default parameters, and all contigs annotated as Caudoviricetes were flagged. In VIBRANT, we used the “VIBRANT_run.py” script with the default parameters, and contigs reported as phages were flagged. In VirSorter2, we used the “virsorter run” pipeline with default parameters, and contigs with a dsDNAphage score > 0.7 were flagged. Next, contigs flagged by two or three tools were selected and processed for quality control using CheckV v1.0.1 [49]. If CheckV reported “no viral genes detected” in a contig, the contig was discarded. If CheckV reported a contig as proviral, the viral segment was extracted from cellular regions and used in downstream analyses. Gene prediction, functional annotation, alignment of the viral contigs, and similarity search to isolated viral genomes were performed and visualized using the ViPTree v4.0 [50] and DiGAlign v2.0 [51] webservers. Further high-sensitivity searches for viral hallmark genes were conducted using SDsearch v0.2.0 [43] with the default parameters against the pfamA hhsuite v35.0 database [52].

### Generation of a reference database from published metagenomic data

We used metagenomic datasets collected by two published studies from Lake Biwa to comprehend the viral genomic diversity in the lake (Fig. S2). The first (Lake Biwa viral contigs, LBVCs) was reconstructed from cellular (0.2–5 μm size fraction) and virion (<0.2 μm) metagenomes collected monthly from June 2016 to February 2017 from the epilimnion and hypolimnion [26]. The second (Lake Biwa viruses, LBVs) was reconstructed from virion (<0.2 μm) metagenomes collected monthly from September 2018 to December 2019 from the epilimnion and hypolimnion [28]. Contigs >10 kb were selected from both datasets and quality-controlled using CheckV, as described above. Finally, the metagenome-derived and SAG-derived viral contigs were pooled and dereplicated using dRep v3.4.2 [53] at the 95% average nucleotide identity (ANI) threshold (-sa 0.95 --ignoreGenomeQuality --S_algorithm ANIn -l 1000, -nc 0.1), which is often used as a threshold to delineate a viral population and to remove the redundancy from the dataset [54, 55]. The resulting 7978 nonredundant viral contigs were used as a reference in read-mapping analyses. Phylogenetic relatedness among the reference contigs was tested by tBLASTx-based homology searching using ViPTreeGen v1.1.3 [50].

### Read mapping analyses

Read mapping and coverage calculation were performed using CoverM v0.6.1 (https://github.com/wwood/CoverM). Raw reads of each SAG were mapped to the corresponding SAG to determine the assembly coverage of the contigs. The raw reads were also mapped to the 7978 reference viral contigs to test their presence in each SAG. In this study, a virus was defined as “detected” when >50% of the viral contig was covered by the mapped reads. In addition, we mapped reads of published metagenomes to the viral contigs to evaluate their spatiotemporal distribution in the lake. We used two datasets: virion (<0.2 μm) metagenomes collected monthly from December 2018 to November 2019 from the epilimnion and hypolimnion [28], and cellular (0.2–5 μm) metagenomes collected monthly from May 2018 to April 2019 [27] (Fig. S2). For metagenomic read mapping, the CoverM option “--min-read-percent-identity 92” was used. Metagenomic read coverages were compared among samples using reads downsampled to 1 million pairs to normalize the sequencing depths.

## Results

### Single-cell amplified genome quality and identification of viral contigs

**Figure 1:**
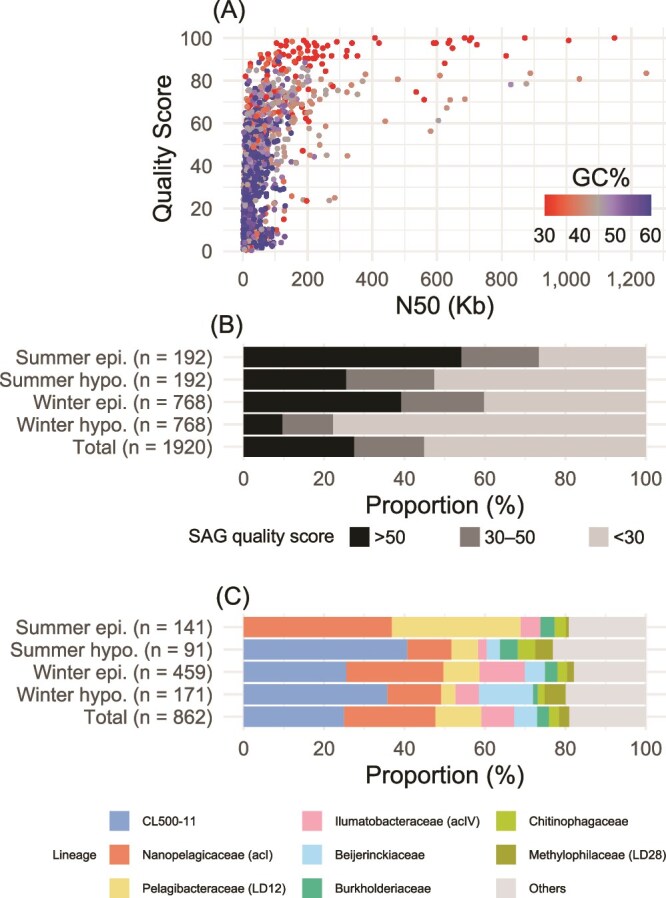
Statistics of single-cell amplified genomes (SAGs). (A) N50, quality score (QS), and GC% distribution of individual SAGs. SAGs with QS < 0 are not shown. (B) Quality distribution of SAGs in each sample and in total. SAGs with QS > 30 were used for the downstream analyses. (C) Phylogenetic composition (family level) of the qualified SAGs in each sample and in total. epi., epilimnion; hypo., hypolimnion.

Among the 1920 sequenced gel beads, 1657 resulted in an assembly with a >1 kb contig. The assembly N50 ranged from 1.0 to 1246.7 kb, and QS ranged from <0 to 100 with a median of 31.75 (Fig. 1A, Table S1). In total, 862 SAGs satisfied our quality standard (QS > 30), including 528 SAGs with QS > 50 (Fig. 1B). The CheckM contamination values of the 862 SAGs averaged 0.6% (median = 0.17%; maximum = 10.5%), and GTDB-tk assigned 643 (74.6%) SAGs to a species (Table S1), ensuring the quality of the SAGs to be used in the downstream analyses. Notably, a 1.15 Mb SAG (LB2301E_00210, genus *Fonsibacter*) was circularity-assembled with a QS of 100. The proportions of the qualified SAGs varied among the samples from 22.3% in the winter hypolimnion to 73.4% in the summer epilimnion (Fig. 1B). Regarding phylogenetic affiliation, eight families (CL500-11, Pelagibacteraceae, Nanopelagicaceae, Ilumatobacteraceae, Burkholderiaceae, Beijerinckiaceae, Chitinophagaceae, and Methylophilaceae) accounted for >80% of all SAGs (Fig. 1C). The summer epilimnion was dominated by Nanopelagicaceae and Pelagibacteraceae, whereas CL500-11 predominated in the other samples.

**Figure 2:**
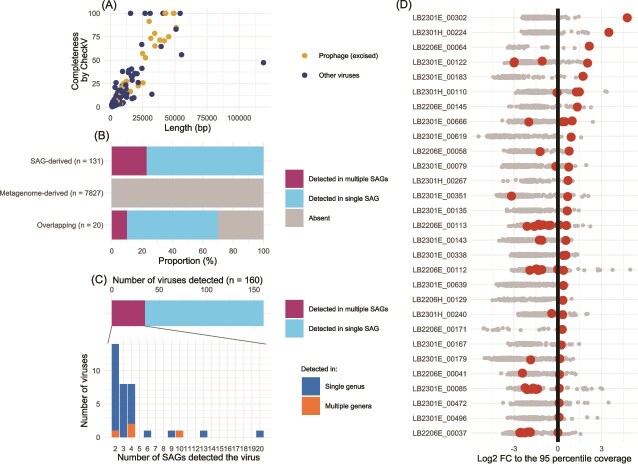
Statistics of detected viruses. (A) Length and completeness distribution of viral contigs assembled in the SAGs. Color indicates whether the contig was detected as a prophage (integrated into a host chromosome). Prophage length is defined as the length of the excised viral region. (B) Proportion of viruses detected in SAGs for each clustering category. Different colors indicate detection in single or multiple SAGs. SAG- and metagenome-derived mean members were dereplicated exclusively from viral contigs originating from SAGs and metagenomic reference sequences, respectively, where those derived from both are classified as overlapping. (C) Distribution of the number of viruses detected in different numbers of SAGs. Top panel, proportions of SAGs in which single and multiple viruses were detected. The total number (*n* = 160) corresponds to the total detected viruses in Fig. 2B. Bottom panel, distribution of multiple detections; colors indicate whether a virus was consistently detected in the same genus. (D) Distribution of contig assembly coverages in each SAG. Coverage was normalized by log2 fold change to the 95th percentile coverage (vertical solid line) for each SAG. Plots indicate individual contigs; viral contigs are indicated by a larger plot in red. Only SAGs with a viral contig with >95th percentile coverage are shown, sorted by normalized coverage of the viral contig.

In total, 176 viral contigs were found in 85 SAGs. CheckV reported that 34 of these were proviruses integrated into host genomes. After the excision of the viral region for a provirus, the viral contigs were 1030–118 892 bp (average = 11 403 bp; median = 5128 bp) in length (Fig. 2A, Table S2). CheckV completeness scores ranged from 0 to 100, with 98 (55.7%) viral contigs showing <10 and 24 viral contigs (13.6%) showing >50. Ten viral contigs were reported as complete (completeness score = 100); five of these were identified as proviruses, and the other five as circular contigs, with the length of the viral region ranging from 15.6 to 54.1 kb (Table S2).

### Viral detection for each single-cell amplified genome through read mapping

The 176 SAG-derived viral contigs were pooled with metagenome-derived contigs and dereplicated at the 95% ANI threshold (Fig. S2). The resulting 7978 nonredundant contigs comprised 131 and 7827 contigs representing clusters generated exclusively from SAG-derived and metagenome-derived contigs, respectively. The other 20 contigs represented clusters including both members. Mapping of SAG raw reads to the viral contigs demonstrated that the 131 SAG-derived viral contigs were detected in at least one SAG, and 30 were detected in multiple SAGs (Fig. 2B). Among the 7827 metagenome-derived viral genomes, 15 were detected, and three were detected in multiple SAGs. Among the other 20 viruses in the overlapping category, 14 were detected, and 2 were detected in multiple SAGs (Fig. 2B).

Among the detected viruses, we screened for those with a higher SAG raw read coverage compared to other assembled contigs in the SAG. We operationally set the 95th percentile of contig coverages in a SAG as the threshold to define high-coverage contigs. We found 29 SAGs with a high-coverage viral contig and inspected their contig coverage distribution. Overall, the coverages were distributed continuously and broadly, with no apparent gap (Fig. 2D). Exceptionally, we observed an extremely high-coverage viral contig in each of the two CL500-11 SAGs, LB2301E_00302 and LB2301H_00224. Their read coverages (555 and 324, respectively) were far higher than the second-highest coverage (55 and 120, respectively) and the 95th percentile coverage (20.6 and 28.8, respectively) observed in each SAG.

Among the 160 viral contigs detected by mapping raw reads of the SAGs (Fig. 2B), 125 were detected in a single SAG, and 35 were detected in multiple SAGs (Fig. 2C). Notably, one virus contig (LB2301E_00219_ctg_540) was detected in 20 SAGs of the same genus of CL500-11. Most other viruses detected in multiple SAGs were also consistently detected in the same genus (Fig. 2C).

**Figure 3:**
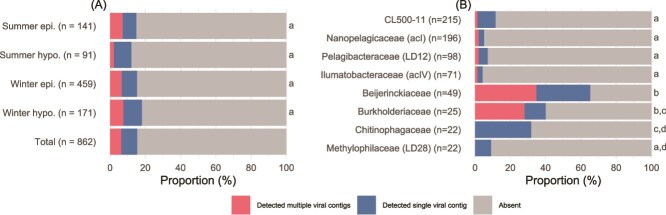
Proportions of SAGs with viral detection. Different colors indicate the detection of single or multiple viral contigs. (A) Proportion in each sample and in total. (B) Proportion in each family. Groups that do not share the same letter on the right side exhibited significant statistical differences (*P* < .05, using Fisher’s exact tests with Benjamini and Hochberg’s false discovery rate correction) in the infection rate (sum of single and multiple detections). epi., epilimnion; hypo., hypolimnion.

**Figure 4:**
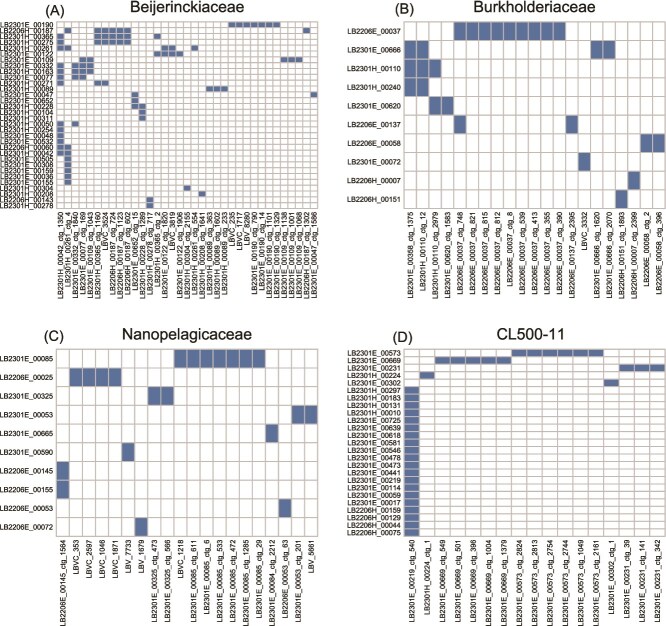
Combinations of SAGs and their detected viral contigs for members of the (A) Beijerinckiaceae, (B) Burkholderiaceae, (C) Nanopelagicaceae, and (D) CL500-11. Horizontal and vertical axes indicate individual viral contigs and SAGs, respectively. Pairs of SAGs and detected viral contigs are indicated by filled boxes.

Viral detection rates among the SAGs in each sample ranged from 12.1% (summer hypolimnion) to 18.1% (winter hypolimnion) (Fig. 3A), and the values in each family ranged from 4.2% (Ilumatobacteraceae) to 65.3% (Beijerinckiaceae) (Fig. 3B). We observed heterogeneous virus–host correspondence within each family (Fig. 4). In the Beijerinckiaceae, for instance, 35 viral contigs were detected from 32 SAGs, and the most widespread virus was detected in less than half of the cells (13 SAGs) (Fig. 4A).

In 55 SAGs, multiple viral contigs were detected (Fig. 3A), implying co-infection by multiple viruses. We inspected the lengths and redundancies of viral marker genes (capsid, terminase, and portal proteins) of the viral contigs detected in the same SAG to determine if they originated from multiple viral genomes or fragments of the same viral genome (Table S3). As a result, one SAG (LB2301E_00122) likely detected contigs of different viral genomes, where marker gene redundancy was observed among multiple >10 kb contigs sharing no homology (Fig. S3). For the other 54 SAGs, the situation was unclear because of fragmented assemblies lacking marker genes (Table S3), except for several cases where the viral contigs originated from the same viral genome that eluded de-replication because the aligned fraction size was lower than the threshold (10%) set in dRep (Fig. S4). Thus, evaluation of the frequency and ecological implications of co-infection warrants a larger-scale study that compensates for the low viral detection rate.

The viral detection rate within each family varied among samples (Fig. S5), but this variation was not statistically supported due to marked differences in the number of SAGs among samples. Although Pelagibacteraceae, Beijerinckiaceae, and CL500-11 harbored one or two species, other dominant families harbored 5 or more species, with a maximum of 15 species within Nanopelagicaceae (Fig. S6).

### Testing the performance of single-cell amplified genome-based host prediction

One could argue that detecting a virus in an SAG does not necessarily indicate a specific interaction and can result from nonspecific attachment of a virus to a cell or random co-encapsulation of a free-living virion and cell during single-cell isolation. In fact, some of our predictions were validated by known virus–host relationships. For example, two viral contigs assembled in the Pelagibacteraceae SAGs (LB2206E_00171_ctg_26 and LB2301E-00765_ctg_133) were relatives of HTVC010P (Fig. S7), a known Pelagibacteraceae virus in marine and freshwater systems [56, 57]. Moreover, most of the viruses detected in multiple SAGs were consistently detected in the same genus (Fig. 2C), precluding random associations. However, in a few cases, the same virus was detected in multiple genera of SAGs (Fig. 2C), and an apparent false positive occurred, in which fragments of viral genomes closely related to known T4-like cyanophage S-SM2 [58] were detected in a Nanopelagicaceae SAG (Figs 4C and S8).

Among the 176 SAG-derived viral contigs, 50 had a short sequence match (>99% identity and >30 bp using BLASTn) to 575 long-read assembled Lake Biwa bacterioplankton MAGs constructed in a previous study [27] (Fig. S2 and Table S2). In 37 out of the 50 cases, the lowest consensus taxonomy of the hit MAGs coincided with the taxonomy of the original SAG at the phylum or lower level (Table S2), supporting the consistency of SAG-based and conventional host prediction methods. However, they were inconsistent at the phylum level in eight SAGs (Table S2), implying contamination of the SAG or MAG. Collectively, although the detection of a virus in a SAG generally reflects a *bona fide* virus–host association, the consistency of the data should be considered due to the possibility of false positives.

### Profiling of the viral distribution in the lake by metagenomic read mapping

**Figure 5:**
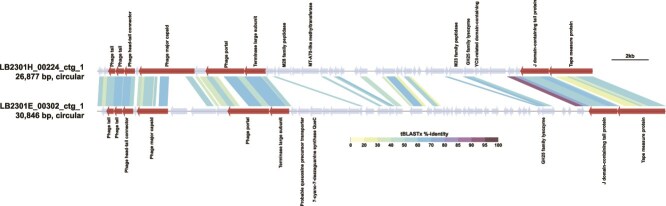
tBLASTx alignment of two circular CL500-11 viruses discovered in this study, LB2301H_00224_ctg_1 and LB2301E_00302_ctg_1. Arrows indicate predicted genes; labels indicate functional annotations; dark red arrow indicates viral structural proteins. Sequences may be inversed or circularly permuted using DiGAlign to show alignment more clearly.

**Figure 6:**
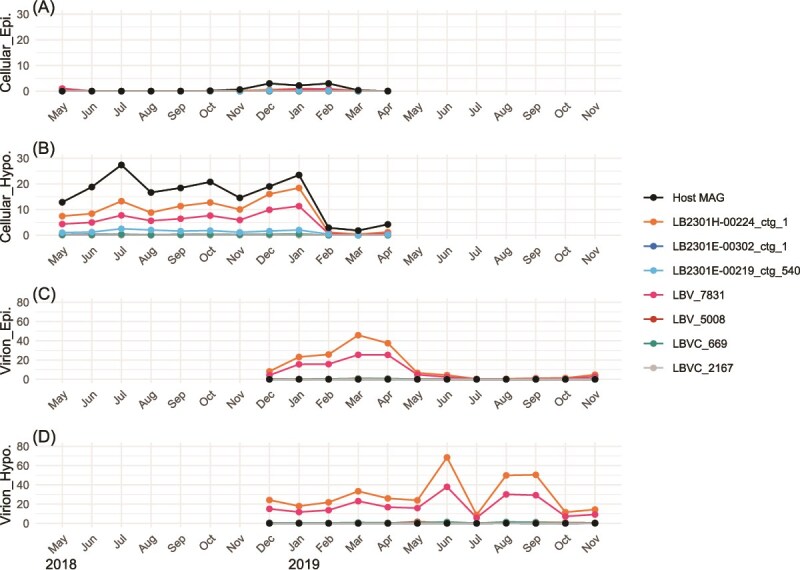
Monthly abundance dynamics of CL500-11 bacteria and their viruses (three contigs featured in the main text and their relatives in the database; Fig. S10) estimated by metagenomic read coverage. Coverages among the samples were normalized by mapping 1 million read pairs downsampled from each sample. Abundances in the (A, B) cellular fraction from May 2018 to April 2019 and (C, D) virion fraction from December 2018 to November 2019 in the epilimnion and hypolimnion, respectively. For the reference host genome, we used the circular CL500-11 long-read assembled metagenome-assembled genomes (MAGs) generated in Lake Biwa [27].

Mapping reads of published metagenomes in the lake revealed that among the 131 dereplicated viral contigs originating exclusively from the SAGs, 73% and 24% were detected (showed >50% coverage breadth) at least in one sample in the cellular and virion metagenomes, respectively (Fig. S9). We next explored the spatiotemporal distribution of CL500-11 viruses by metagenomic read mapping. We selected two high-coverage viral contigs (LB2301E_00302_ctg_1 and LB2301H_00224_ctg_1) (Fig. 2D) and a viral contig detected in 20 CL500-11 SAGs (LB2301E_00219_ctg_540) (Fig. 2C). Notably, the two high-coverage contigs were both circularly assembled and shared synteny (Fig. 5). By querying these contigs against the metagenome-derived viral contigs, we identified four contigs (LBV_5008, LBV_7831, LBVC_2167, and LBVC_669) that shared synteny to the two circular viruses (Fig. S10). A total of seven viral contigs and a long-read assembled circular MAG of the host (CL500-11) recovered previously from the lake [27] were used together as a reference for metagenomic read mapping. LB2301E_00302_ctg_1 and LBV_7831 showed higher read coverage than other viral contigs in the hypolimnion and during the mixing period in both the cellular and virion fractions (Fig. 6). LB2301E_00219_ctg_540 was subsequently abundant but showed disproportionally higher coverage in the cellular fraction than in the virion fraction (Fig. 6).

## Discussion

### Single-cell amplified genome-gel recovered high-quality single-cell amplified genomes representing the lake bacterial community

Although limited assembly quality is a major problem in single-cell genomics [21, 23, 59], we generated as many as 862 qualified SAGs (Fig. 1A and B and Table S1) with a circularly assembled bacterial genome, demonstrating a good performance of SAG-gel technology. Their high quality is also attributable to the dominance of low-GC bacteria (e.g. Pelagibacteraceae and Nanopelagicaceae), facilitating less biased whole-genome amplification [22, 60]. Indeed, our high-QS SAGs were enriched in low-GC genomes (Fig. 1A). The phylogenetic composition of the SAGs was generally in agreement with the known composition of the bacterial community in the lake; members of Pelagibacteraceae and Nanopelagicaceae generally dominate across depths and seasons, whereas CL500-11 shows a preference for the hypolimnion and is absent in the summer epilimnion [27, 29]. The occurrence of CL500-11 in the winter epilimnion can be explained by the dispersal of cells by the onset of winter vertical mixing [32]. The other dominant members (Ilumatobacteraceae, Burkholderiaceae, Beijerinckiaceae, Chitinophagaceae, and Methylophilaceae) are also abundant bacterioplankton lineages in Lake Biwa and other freshwater lakes [27, 61–63]. Overall, our SAGs represented the diverse bacterioplankton community in the lake, allowing detailed single-cell-resolution investigation within and between lineages.

### Viral signals in single-cell amplified genomes revealed high viral diversity and novel virus–host pairs

Although we identified 176 viral contigs from the SAGs, most were fragmented (Table S2), implying the presence of unassembled viruses. For more comprehensive viral detection by read mapping analysis, we leveraged viral contigs from published Lake Biwa metagenomes (Fig. S2). However, the SAG- and metagenome-derived viral contigs were rarely clustered into the same group at the 95% ANI threshold, and almost all metagenome-derived contigs were not detected by SAG read mapping (Fig. 2B), indicating their absence in the sequenced cells. Among the exclusively SAG-derived viruses, 73% and 24% were detected by cellular and virion metagenomic read mapping, respectively (Fig. S9). Their metagenomic detection indicates their presence in the samples, although they were unassembled in the metagenomes, presumably due to their low abundance or high genomic microdiversity, which hinders metagenomic assembly [64–66]. By contrast, SAG-derived viruses not detected by metagenomic read mapping indicate their absence in the metagenomic samples, possibly because of the temporal shift of the viral community in the lake, as the metagenomes were collected 3–4 years prior to the collection of our SAGs. Collectively, the gap between the SAG and metagenomic viromes indicated the viral community in the lake to be highly diverse.

An important achievement of this study is the identification of novel virus–host pairs by the detection of viral signals in SAGs, which is unprecedented in a freshwater lake. Although large-scale viral metagenomic surveys in freshwater lakes routinely recover thousands of viral genomes [26, 28, 30, 67], the hosts of most of these viruses are unknown, which limits understanding of their ecology. Furthermore, host prediction relies mainly on gene phylogeny, which does not resolve the host to a low taxonomic level. For example, the actinobacterial *whiB* gene contributes a significant portion of the host prediction in freshwater viromes [28, 30, 68], but it predicts the host only to the phylum level, which limits ecological interpretations, given the broad diversity of *Actinobacteria* in freshwater ecosystems [31, 62, 69]. Our results linked genomes of viruses and hosts at the species resolution (Fig. S6 and Table S2), including ecologically important viral members infecting ubiquitous and abundant bacterioplankton lineages, as demonstrated by the discovery of CL500-11 virus discussed later.

We characterized high-coverage viral contigs to differentiate viruses within a lytic cycle (i.e. high copy number in a cell). However, as reported previously [18], the assembly coverage of the contigs in an SAG exhibited broad and continuous distribution (Fig. 2D) due to the bias introduced by whole-genome amplification [22, 60]. Thus, the assembly coverage did not enable objective definition of active viruses, except for the two CL500-11 circular viruses, as discussed below.

### Infection rate of the lake bacterioplankton community

In the present study, none of the abundant (*n* > 5) host species showed a 100% viral detection rate (Fig. S6), indicating the mobility of the viruses and the absence of a persistent provirus. Thus, their “infection” can be defined based on their detection by read mapping, regardless of whether they are provirus or not. The infection rates in our samples (12.1%–18.1%; Fig. 3A) were higher than those of visibly infected cells (0.9%–4.1%) previously reported by transmission electron microscopy in Lake Biwa [6, 70]. The higher rate obtained in the present study suggests the high sensitivity of our read mapping–based approach to detect viral associations without virion formation. Nevertheless, our viral infection rates were lower than those in prior single-cell genomics studies (26% to >60%) [15, 16, 18, 19]. This gap is attributable to differences in the diversity of the host community because these studies targeted low-diversity communities or small numbers of taxa. It is also possible that our viral infection rate was underestimated because of our strict criteria for the viral contig identification. However, relaxing the criteria would introduce more mobile genetic element (MGE)–like sequences due to the presence of virus-like genes in some MGEs, as is the case of LB2301E_00219_ctg_540 discussed below. Therefore, leveraging the high-quality assembly of our SAGs, we applied conservative criteria by taking the consensus of three virus identification tools (i.e. geNomad, VIBRANT, and VirSorter2) to limit our analysis to confidently identified dsDNA phages. Notably, the viral infection rate of Pelagibacteraceae (7.1%; Fig. 3B) was comparable to those determined for marine Pelagibacteraceae and their abundant phages by fluorescence *in situ* hybridization [8].

### Contrasting viral defense strategies of oligotrophs and copiotrophs

The viral infection rate showed no significant variance across four samples from different seasons and water layers (Fig. 3A) but showed significant variance among host lineages (Fig. 3B), suggesting that susceptibility to viral infection depends on the host lineage. We found copiotrophic bacterial lineages had higher viral infection rates than oligotrophic lineages. Oligotrophs (i.e. K-strategists) are characterized by slow growth and persistent dominance in pelagic open water systems by adapting to stable and resource-poor conditions. By contrast, copiotrophs (i.e. r-strategists) are characterized by rapid growth and opportunistic dominance under resource-rich conditions and have a versatile metabolism, enabling their survival under competitive conditions [61, 71, 72]. Specifically, members of the Pelagibacteraceae (also known as LD12 in freshwater systems), Nanopelagicaceae (acI), Ilumatobacteraceae (acIV), and Methylophilaceae (LD28), which are typical oligotrophs in freshwater ecosystems [61, 73], showed lower viral infection rates than the Burkholderiaceae and Chitinophagaceae (Fig. 3B), typical copiotrophs dominating under resource-rich conditions such as algal blooms [61, 73]. The highest viral infection rate was for Beijerinckiaceae, most of which were affiliated with a species of *Methylocystis* (Figs 3B and S6), which are aerobic methane-oxidizing bacteria [74]. They have been regarded to be copiotrophs in the lake based on their nonpersistent occurrence [27, 29], likely reflecting the highly spatially and temporally heterogeneous methane concentration in the lake [75]. Although the taxonomic resolution of the comparative analysis was limited to the family level due to small and variable sample sizes, oligotrophs consistently showed low infection rates even at the species resolution (Fig. S6), suggesting the universality of this trend within these families.

**Figure 7:**
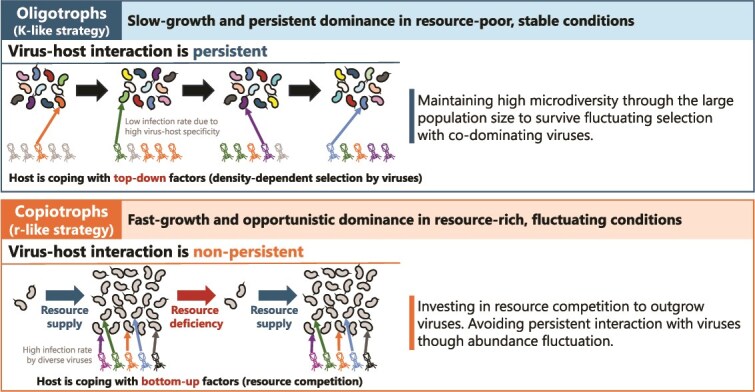
Contrasting viral defense strategies of oligotrophs and copiotrophs proposed based on the results. Oligotrophs cope with stronger density-dependent virus–host selection due to persistent codominance with their viruses. Genome microdiversification to diversify virus–host specificity is an effective strategy for oligotrophs due to their large population size, which explains the low infection rate of their population. By contrast, copiotrophs undergo weaker density-dependent virus–host selection via nonpersistent interactions with viruses due to their fluctuating abundance depending on resource availability. Therefore, the strategy of copiotrophs is to invest more in resource competition to outgrow viruses, rather than coping with the virus–host arms race, which explains the high infection rates of copiotrophs by diverse viruses.

Intriguingly, the high viral infection rates of copiotrophs resulted not from the domination of a single virus but from collective infection by diverse viruses (Fig. 4A and B). Infection by diverse viruses suggests weak density-dependent virus–host selection, because a lower infection rate (resistant hosts are selected) or occupancy of a few viruses (viruses breaking host resistance are selected) is expected under strong density-dependent virus–host selection. We argue that the growth of copiotrophs is primarily controlled by resource availability, making their interaction with viruses nonpersistent. Thus, the host population collapses due to resource deficiency before density-dependent virus–host selection occurs. The strategy of the host is to invest more in resource competition to outgrow rather than defend against viruses (Fig. 7). This strategy is analogous to the greater vulnerability of freshwater copiotrophs to protistan grazing because they have larger cells and compensate for grazing loss with a higher growth rate than that of oligotrophs [73, 76]. Overall, our results support the notion that copiotrophs are primarily coping with bottom–up factors (competition for resources) and that fluctuations in their abundance prevent selective interactions with top–down factors (viral lysis and protistan grazing).

Oligotrophs exhibited a low viral infection rate (Fig. 3B) despite their persistence, indicating resistance to viral infection. Streamlined genomes of oligotrophs harbor fewer genetic defense systems such as CRISPR/Cas [31, 71, 72], and their viral defense is thought to be achieved by a high intraspecies microdiversity [2, 31, 77]. For example, hypervariable region 2 (HVR2) of the Pelagibacteraceae is enriched for glycosyltransferases and is thought to be responsible for generating the diversity of cell surface glyco-structures to evade phage recognition [78–81]. Indeed, a pangenome graph of our Pelagibacteraceae SAGs demonstrated that their HVR2 regions were nearly unique to individual cells (Fig. S11), as also reported in a recent study of marine Pelagibacteraceae [82]. Unlike the fluctuating abundance of copiotrophs, oligotrophs are less likely to be affected by population bottlenecks and thus maintain their genetic microdiversity [27]. High microdiversity has been reported for viruses of oligotrophs, likely as a consequence of persistent fluctuating selection [83–86]. Overall, our results highlight the contrasting viral defense strategies of oligotrophs and copiotrophs, inviting further investigation of their interactions with viruses (Fig. 7).

### Single-cell amplified genomes revealed dominant CL500-11 viruses and their infection dynamics

This study is the first to identify a virus infecting CL500-11 (phylum Chloroflexi), an uncultured bacterioplankton lineage dominant in the oxygenated hypolimnion of deep freshwater lakes globally [33, 34, 41]. CL500-11 is persistently dominant in the hypolimnion during the stratification period [32, 34], hinting at its oligotrophic lifestyle. Besides, their abundance decreases with the collapse of the thermocline during winter mixing [32, 34], prompting the investigation of viruses as potential mortality factors. However, the viruses eluded exhaustive metagenomic efforts to find them using sequence matching, nucleotide frequencies, gene phylogeny, and co-occurrence with potential hosts [26, 28]. Here, we detected 17 viral contigs from 25 CL500-11 SAGs, among which the same viral contig was detected in 20 SAGs, and 14 viral contigs were detected in three SAGs (Fig. 4D). The other two viral contigs were circularly assembled, shared synteny, and were detected in two different CL500-11 cells collected in winter (Figs 4D and 5). Together with their relatives in the reference virome (Fig. S10), these viruses showed a spatiotemporal distribution following that of the host in the metagenomes (Fig. 6), suggesting them to be *bona fide* CL500-11 viruses.

The two circular CL500-11 viruses exhibited far the highest assembly coverage than other chromosomal contigs in the SAG (Fig. 2D). Such an extreme viral contig coverage in an SAG was not observed in a prior study [18] and suggests lytic activity and a high burst size. Moreover, LB2301H_00224_ctg_1, one of the circular viruses, showed high metagenomic read coverages in the cellular and virion fractions in the hypolimnion throughout the water stratification period (Fig. 6), indicating their active replication in a cell and release of virions to the water column. Intriguingly, their metagenomic read coverage in the cellular fraction was close to that of their host in the hypolimnion at the end of stratification period (Fig. 6), although the virus was detected only in one SAG among the 215 CL500-11 SAGs analyzed (Fig. 4D). Collectively, our results suggest that LB2301H_00224_ctg_1 is an abundant and active lytic virus with a high burst size that infects a minor fraction (<1%) of the host population. Of note, previous studies in Lake Biwa observed CL500-11-like (large, curved) cells full of virions inside using transmission electron microscopy [6, 70]. As reported in other oligotrophs, genomic microdiversity may underlie virus–host specificity in this system. Indeed, the microdiversity of CL500-11 was comparable (~40 000 SNVs/Mb) to those of other oligotrophs according to metagenomic read mapping [27]. In summary, the combination of SAG and metagenomic data revealed one of the most quantitatively significant virus–host pairs discovered in freshwater systems and the marked heterogeneity of their interactions, which has been overlooked in metagenomics studies.

Another CL500-11 viral contig, LB2301E_00219_ctg_540, was the most prevalent viral contig in this study, being detected in 20 (9.3%) out of the 215 CL500-11 SAGs (Figs 2C and 4D). The contig showed detectable metagenomic read coverage in the cellular fraction but was absent from the virion fraction (Fig. 6). It encoded an integrase and showed homology to a portion (inserted between tRNAs) of a CL500-11 MAG assembled in a previous study, in which complex structural variants were identified by long-read metagenomic read mapping [27] (Fig. S12). LB2301E_00219_ctg_540 is dominated by repetitive proteins and harbors only one viral marker gene (a phage head morphogenesis protein) in nested structural variants (Fig. S12). Collectively, we concluded that LB2301E_00219_ctg_540 is an MGE identified as a viral contig based on the presence of an auxiliary viral structural-like protein. Although MGE is beyond the scope of this study, the dissemination of the integrated MGE within the persistent and microdiverse host population is intriguing. We propose that the function of the enriched genetic structural variant (Fig. S12) is the key to understanding the mechanism underlying its persistent codominance with its host.

## Conclusion

By leveraging 862 SAGs and a published metagenomic dataset, we revealed novel and heterogeneous virus–host associations at single-cell resolution in Lake Biwa. Notably, we discovered multiple CL500-11 viruses, which had eluded exhaustive metagenomic efforts to find them. Comparative analyses among the dominant host lineages highlighted contrasting viral defense strategies between oligotrophs and copiotrophs and supported the hypothesis that the persistence of virus–host interactions is the key to understanding the mechanisms underlying their co-existence (Fig. 7). Our study suggested two further steps to validate the hypothesis in the future. The first is to comprehensively compare the infection rate between oligotrophs and copiotrophs in different environments. As our observations only covered the dominant bacterioplankton lineages in a lake ecosystem (Fig. 3B), other environments and rarer lineages should also be investigated to test the generality of the proposed theory (Fig. 7). The second is to target a broader range of viruses. Our analytical pipeline primarily targets dsDNA-tailed bacteriophage (Caudoviricetes), but there are other types of viruses that infect prokaryotes, such as nontailed dsDNA, single stranded DNA, and RNA viruses [87]. Furthermore, with the rapid accumulation of nucleotide data and advances in bioinformatics technology, new high-taxonomic groups of viruses are still being discovered in recent years [88, 89]. Whether the addition of more virus types in the analysis will even support our hypothesis (Fig. 7), or reveal other forms of virus–host interaction, remains to be seen in future work. Regarding the single-cell genomics technology, overcoming the issue of coverage bias, which resulted in fragmented viral genomic assemblies (Fig. 2A) and hindered the identification of active lytic viruses (Fig. 2D), remains a major challenge. The application of novel DNA amplification techniques, such as primary template–directed amplification [90], is anticipated to be essential, as going beyond metagenomic resolution will be a prerequisite in future microbial ecology.

## Supplementary Material

LBSAG_Virus_ms_SupplementaryInfo_ycaf086

TableS1_ycaf086

TableS2_ycaf086

TableS3_ycaf086

## Data Availability

The raw sequencing reads for the 862 SAGs are available under BioProject ID PRJDB18380. The nucleotide fasta files of the assembled 862 SAGs are available at https://doi.org/10.6084/m9.figshare.26243444
