# Supplementary material for: Contrasting defense strategies of oligotrophs and copiotrophs revealed by single-cell-resolved virus–host pairing of freshwater bacteria
Source: ISME Commun. 2025 May 21;5(1):ycaf086. doi: 10.1093/ismeco/ycaf086 (PMC12143481; doi:10.1093/ismeco/ycaf086)
Supplement: LBSAG_Virus_ms_SupplementaryInfo_ycaf086 [file lbsag_virus_ms_supplementaryinfo_ycaf086.pdf]

Supplementary Information for:

Contrasting defense strategies of oligotrophs and copiotrophs revealed by single-cell-resolved virus–host pairing of freshwater bacteria

Yusuke Okazaki*, Yohei Nishikawa, Ryota Wagatsuma, Haruko Takeyama, Shin-ichi Nakano

*Corresponding author:
Yusuke Okazaki
Institute for Chemical Research, Kyoto University, Gokasho, Uji, Kyoto, 611-0011, Japan
E-mail: okazaki.yusuke.e31@kyoto-u.jp

This PDF file includes:
Figures S1 to S12
Captions for Tables S1 to S3

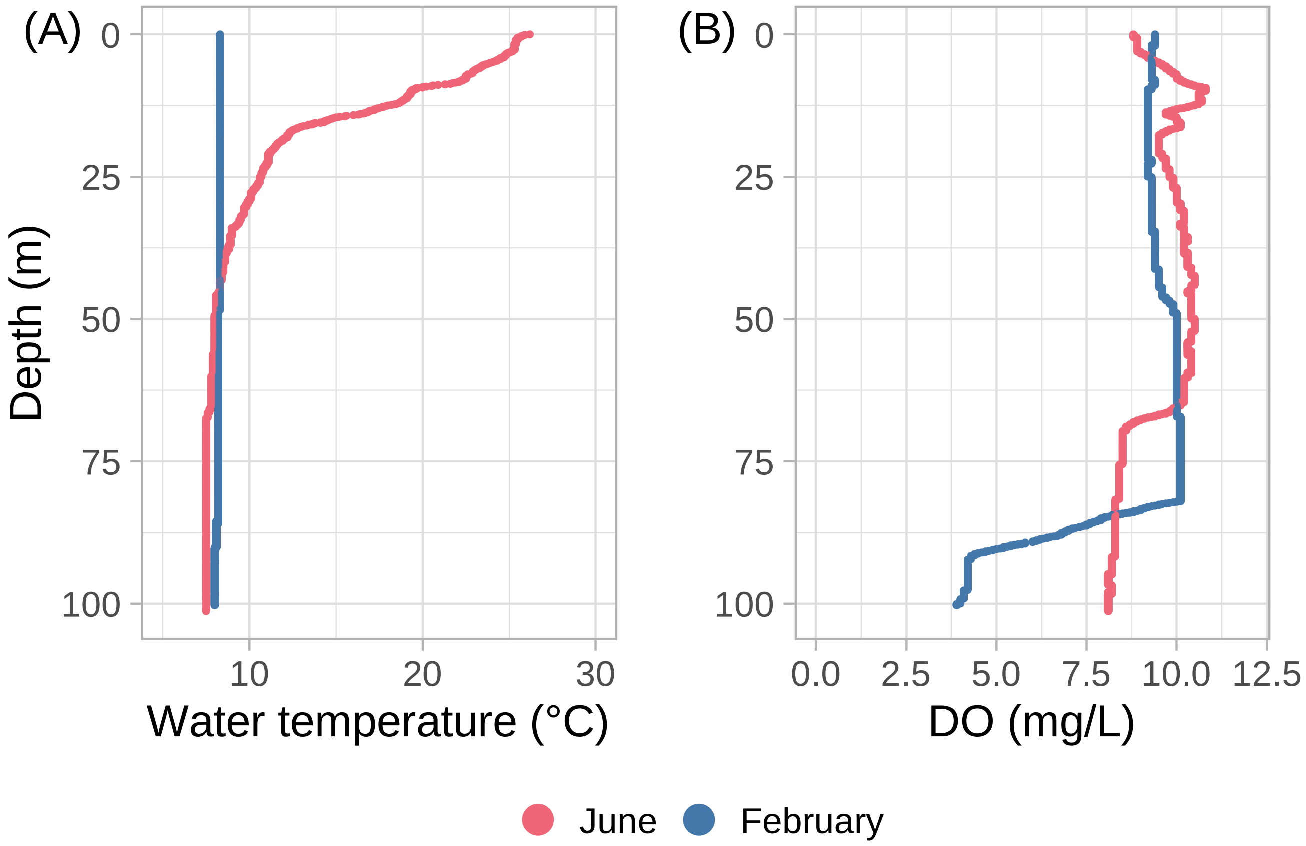

Fig. S1. Vertical profiles of (A) water temperature and (B) dissolved oxygen (DO) concentration at the time of sampling (June 29, 2022 and February 1, 2023).

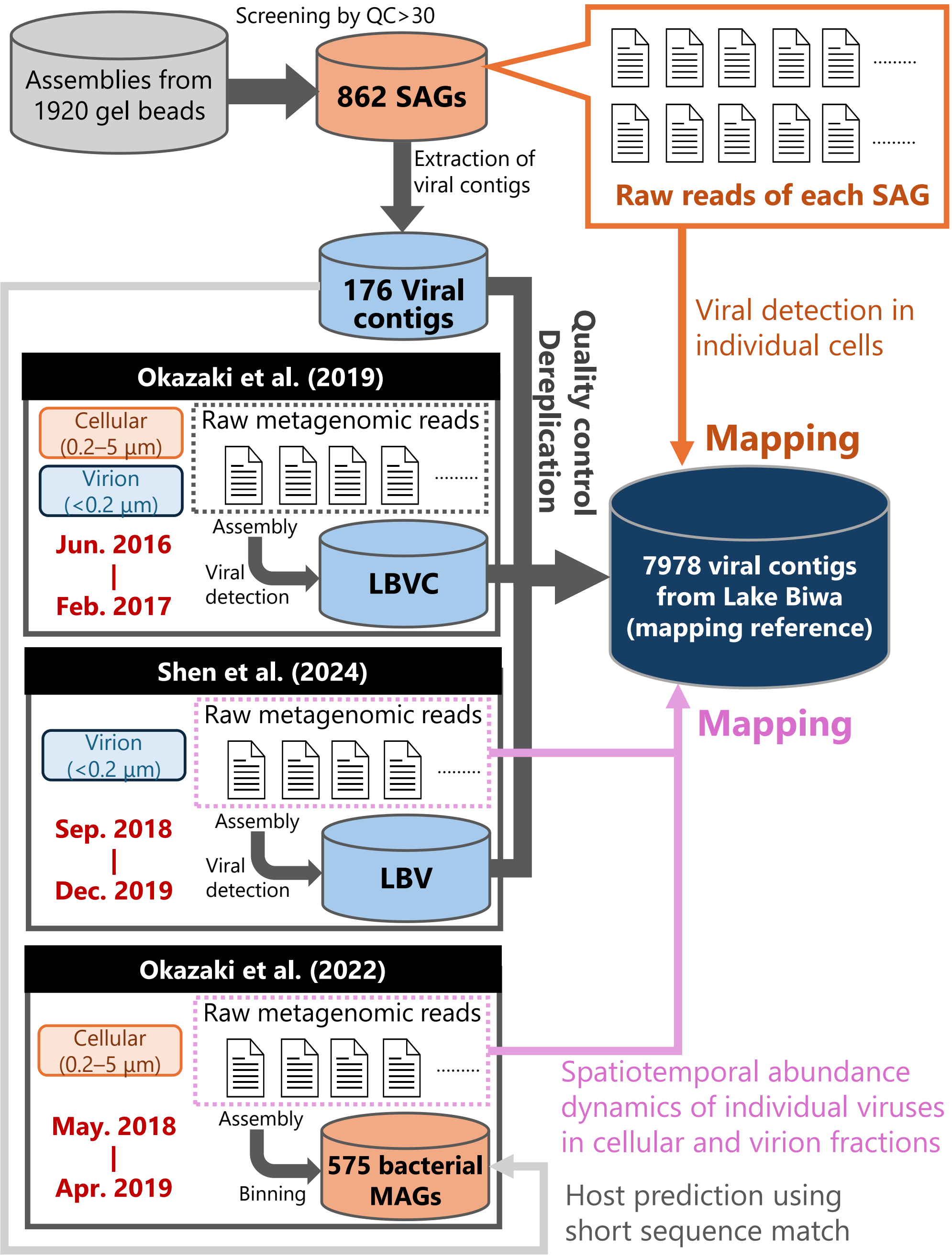

Fig S2. Data structure and analytical pipeline in the present study. The 176 viral contigs assembled in the SAGs, Lake Biwa viral contigs (LBVC) generated in Okazaki et al. (2019), and Lake Biwa viruses (LBV) generated in Shen et al. (2024) were pooled, quality controlled, and dereplicated to create a non-redundant set of viral contigs, which was used in two mapping-based analyses. The first is mapping of raw reads of each SAG aiming to detect the viruses in the individual cells. The second is mapping of raw metagenomic reads collected monthly from the virion (Shen et al., 2024) and cellular (Okazaki et al., 2022) fractions aiming to reveal spatio-temporal abundance dynamics of the viruses. In addition, we used the 575 long-read assembled bacterial MAGs generated in Okazaki et al. (2022) to predict the host of the 176 SAG-derived viral contigs using a conventional sequence matching approach.

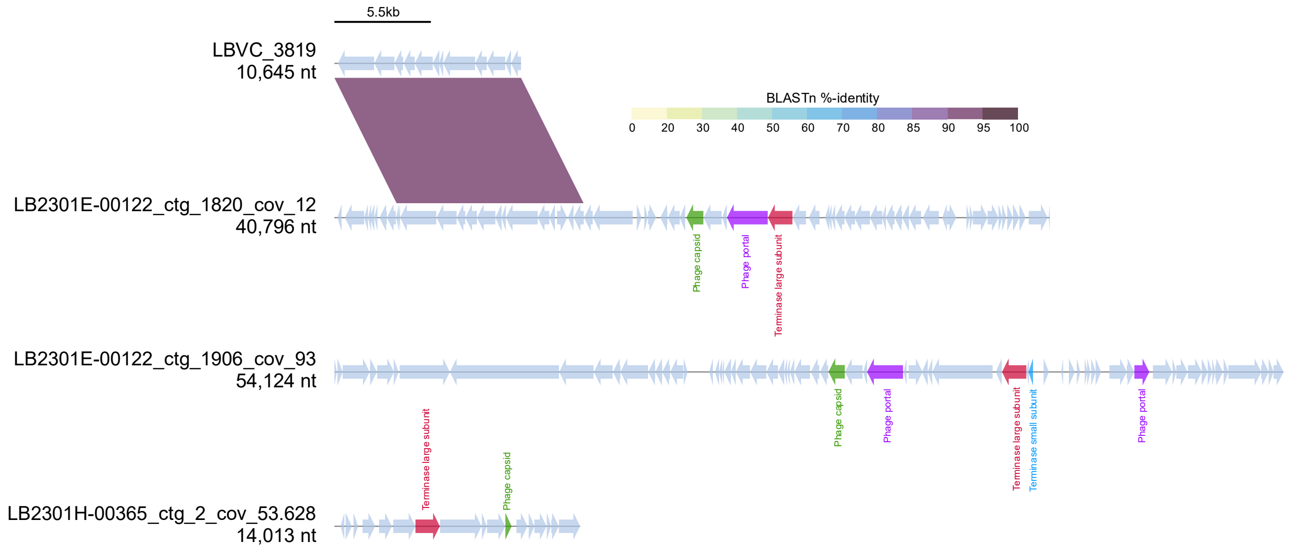

Fig S3. BLASTn-alignment of four viral contigs detected in the same SAG (LB2301E-00122). Arrows indicate predicted genes; colors indicate viral structural genes. Three viral contigs with overlapping structural proteins showed no homology, indicating the detection of multiple viral genomes from a single cell. The sequences may be inversed using DiGAlign to show alignment more clearly.

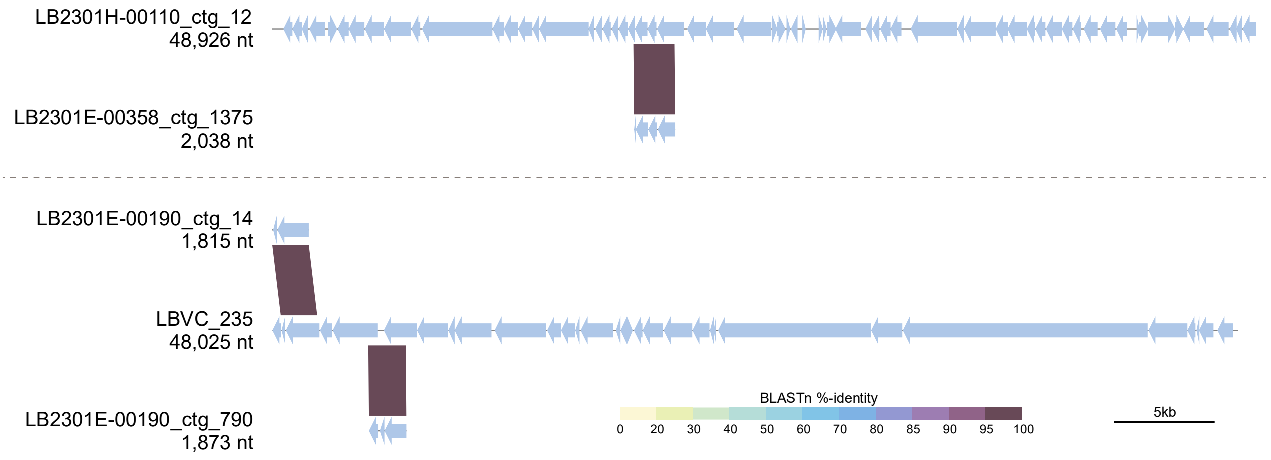

Fig. S4. BLASTn alignment of viral contigs co-detected in two SAGs, LB2301H-00110 (top two sequences) and LB2301E-00190 (bottom three sequences). Short contigs are aligned to a much longer contig, suggesting that they originated from the same viral genome but eluded de-replication because the aligned fraction size was lower than the threshold (10%) set in dRep software. Sequences may be inversed using DiGAlign to show alignment more clearly.

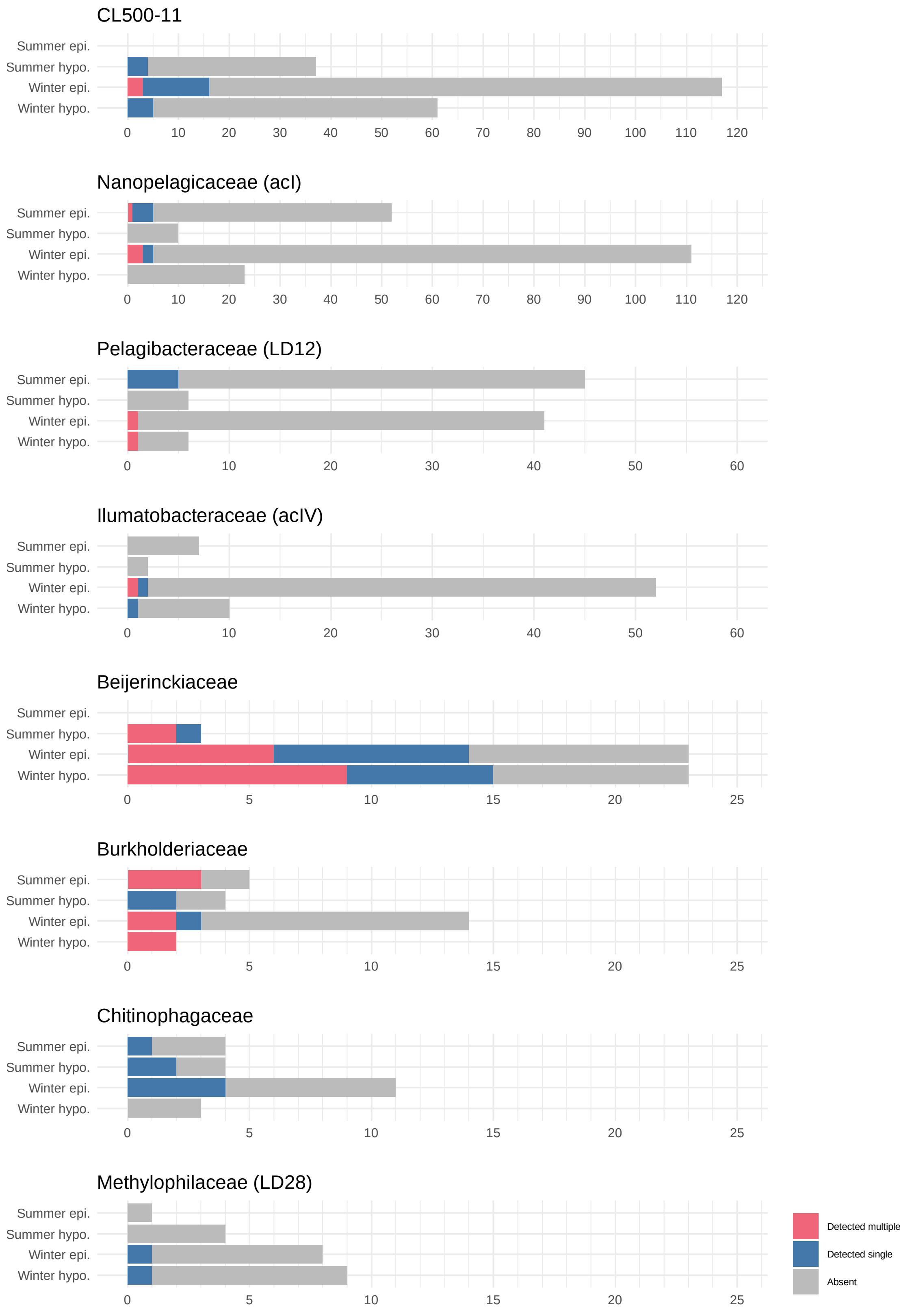

Fig. S5. Numbers of SAGs with viral detection for each sample in each family. The detection of single or multiple viral contigs is indicated by different colors.

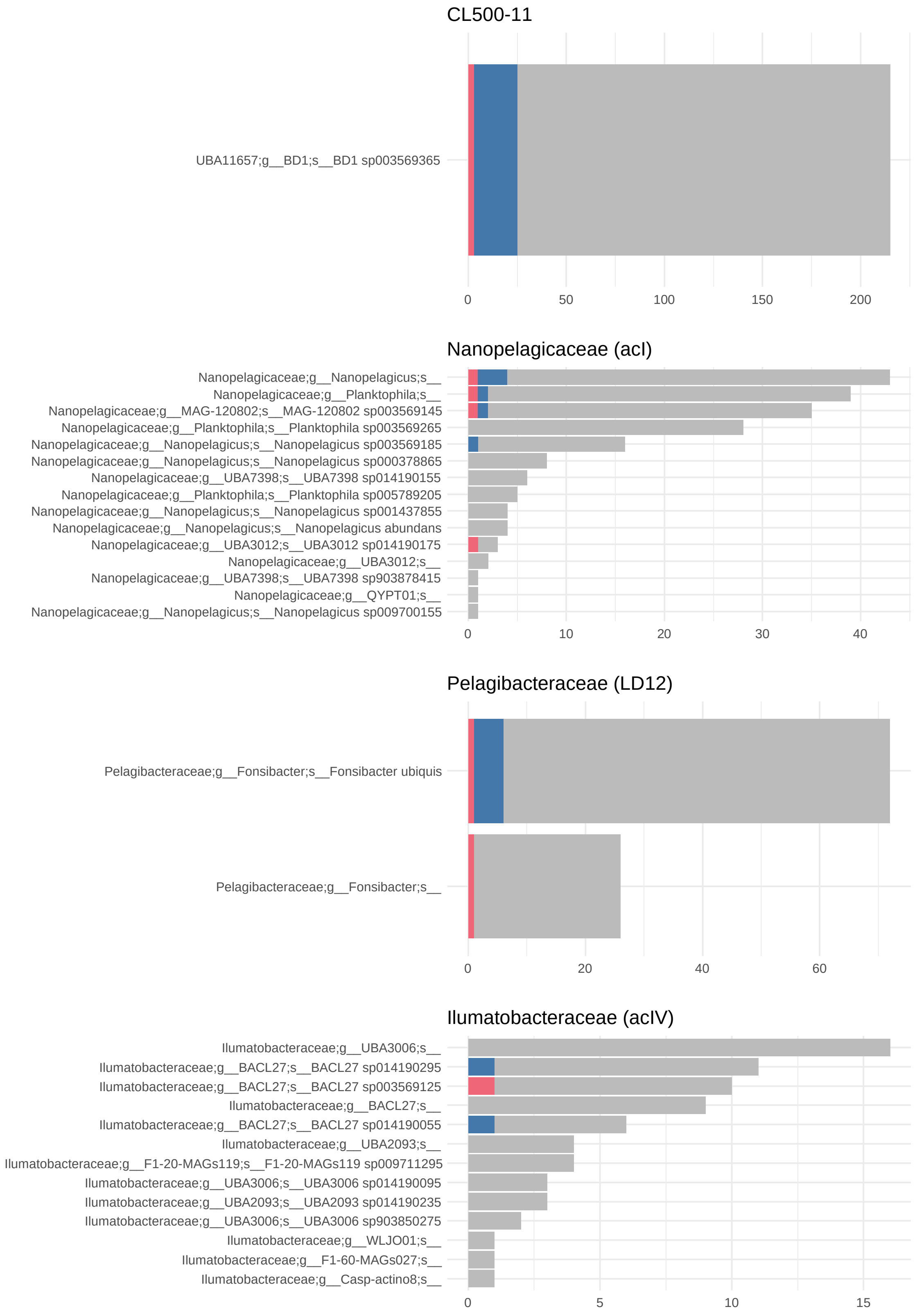
CL500-11
UBA11657;g__BD1;s__BD1 sp003569365
0
50
100
150
200
Nanopelagicaceae (acI)
Nanopelagicaceae;g__Nanopelagicus;s__
Nanopelagicaceae;g__Planktophila;s__
Nanopelagicaceae;g__MAG-120802;s__MAG-120802 sp003569145
Nanopelagicaceae;g__Planktophila;s__Planktophila sp003569265
Nanopelagicaceae;g__Nanopelagicus;s__Nanopelagicus sp003569185
Nanopelagicaceae;g__Nanopelagicus;s__Nanopelagicus sp000378865
Nanopelagicaceae;g__UBA7398;s__UBA7398 sp014190155
Nanopelagicaceae;g__Planktophila;s__Planktophila sp005789205
Nanopelagicaceae;g__Nanopelagicus;s__Nanopelagicus sp001437855
Nanopelagicaceae;g__Nanopelagicus;s__Nanopelagicus abundans
Nanopelagicaceae;g__UBA3012;s__UBA3012 sp014190175
Nanopelagicaceae;g__UBA3012;s__
Nanopelagicaceae;g__UBA7398;s__UBA7398 sp903878415
Nanopelagicaceae;g__QYPT01;s__
Nanopelagicaceae;g__Nanopelagicus;s__Nanopelagicus sp009700155
0
10
20
30
40
Pelagibacteraceae (LD12)
Pelagibacteraceae;g__Fonsibacter;s__Fonsibacter ubiquis
Pelagibacteraceae;g__Fonsibacter;s__
0
20
40
60
Ilumatobacteraceae (acIV)
Ilumatobacteraceae;g__UBA3006;s__
Ilumatobacteraceae;g__BACL27;s__BACL27 sp014190295
Ilumatobacteraceae;g__BACL27;s__BACL27 sp003569125
Ilumatobacteraceae;g__BACL27;s__
Ilumatobacteraceae;g__BACL27;s__BACL27 sp014190055
Ilumatobacteraceae;g__UBA2093;s__
Ilumatobacteraceae;g__F1-20-MAGs119;s__F1-20-MAGs119 sp009711295
Ilumatobacteraceae;g__UBA3006;s__UBA3006 sp014190095
Ilumatobacteraceae;g__UBA2093;s__UBA2093 sp014190235
Ilumatobacteraceae;g__UBA3006;s__UBA3006 sp903850275
Ilumatobacteraceae;g__WLJO01;s__
Ilumatobacteraceae;g__F1-60-MAGs027;s__
Ilumatobacteraceae;g__Casp-actino8;s__
0
5
10
15

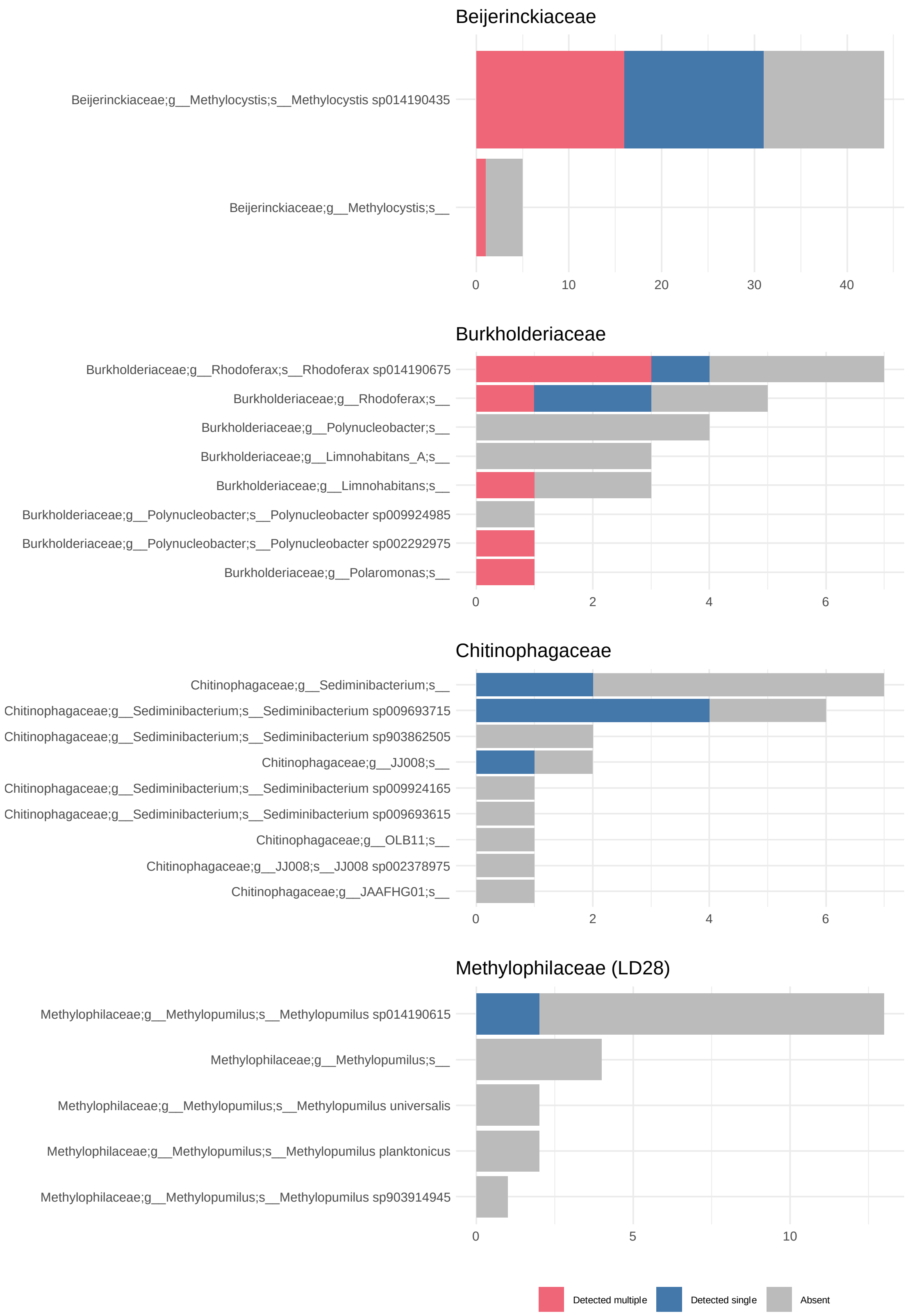

Fig. S6. Numbers of SAGs with viral detection for each species in each family. The detection of single or multiple viral contigs is indicated by different colors.

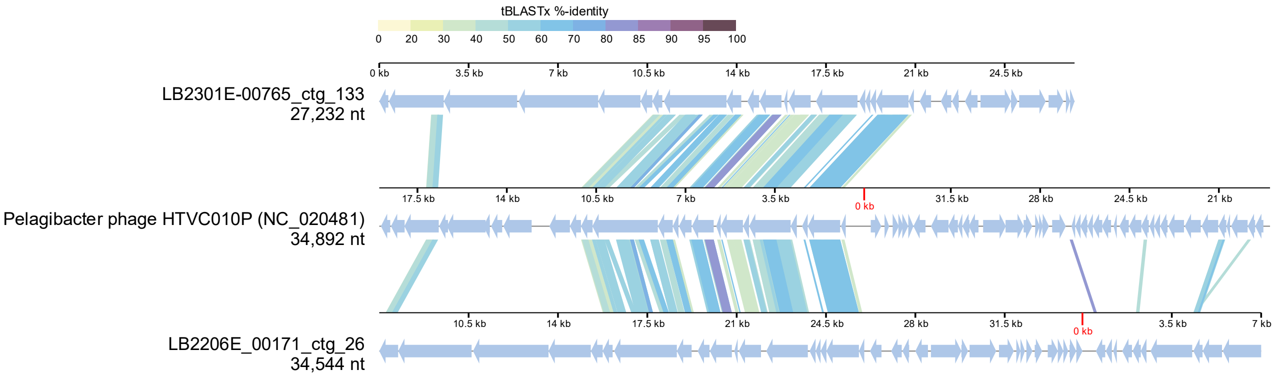

Fig. S7. tBLASTx alignment of the Pelagibacter phage HTVC010P and its related viral contigs (LB2301E_00765_ctg_133 and LB2206E_00171_ctg_26) recovered from Pelagibacteraceae SAGs. Sequences may be inversed or circularly permuted using DiGAlign to show alignment more clearly.

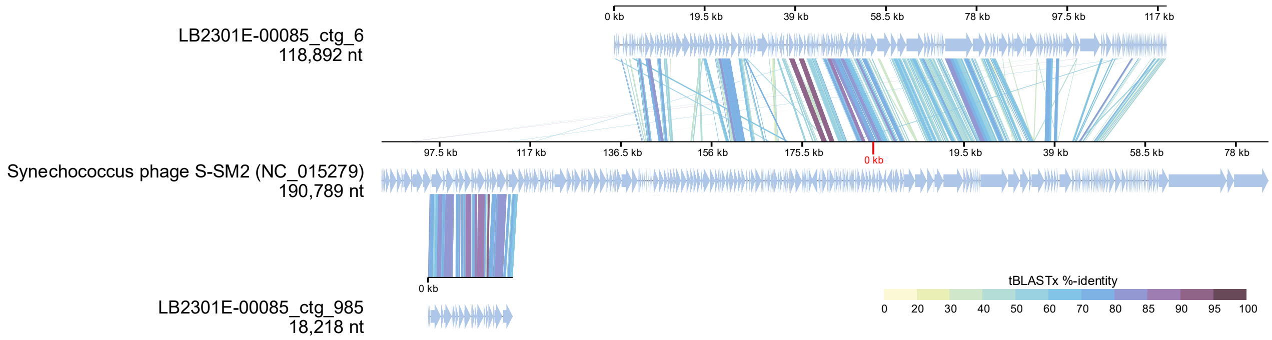

Fig. S8. tBLASTx alignment of *Synechococcus* phage S-SM2 and its related viral contigs recovered from LB2301E-00085, an actinobacterial SAG. Sequences may be inversed or circularly permuted using DiGAlign to show alignment more clearly.

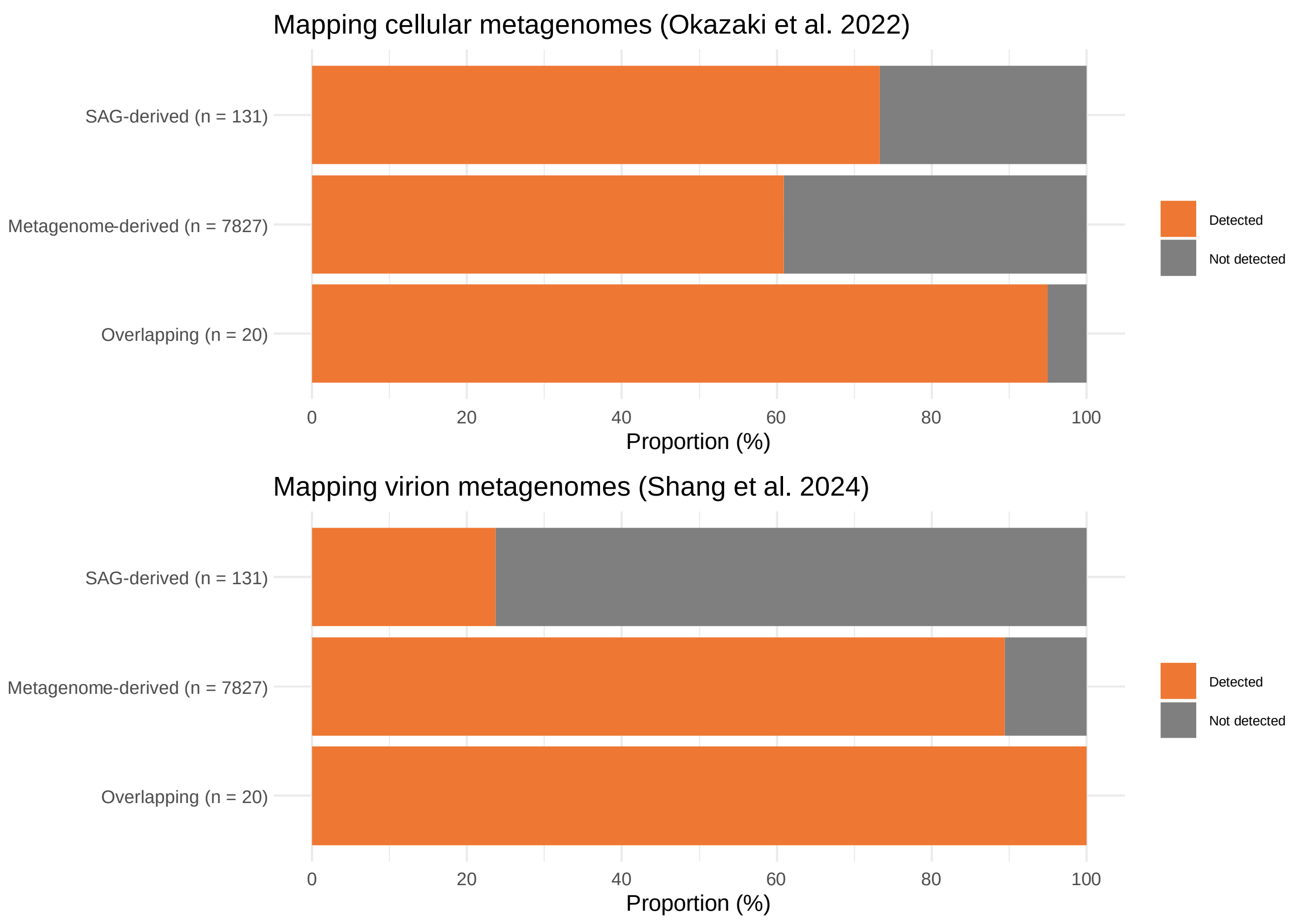

Fig S9. Proportions of viruses detected by metagenomic read mapping in cellular and virion metagenomes. If one of the 24 metagenomic samples in each size fraction showed a > 50% mapping breadth in a contig (i.e., > 50% of a contig was covered by mapped reads), the virus was regarded as detected in the size fraction. Viruses are grouped into three categories, as in Fig. 2B.

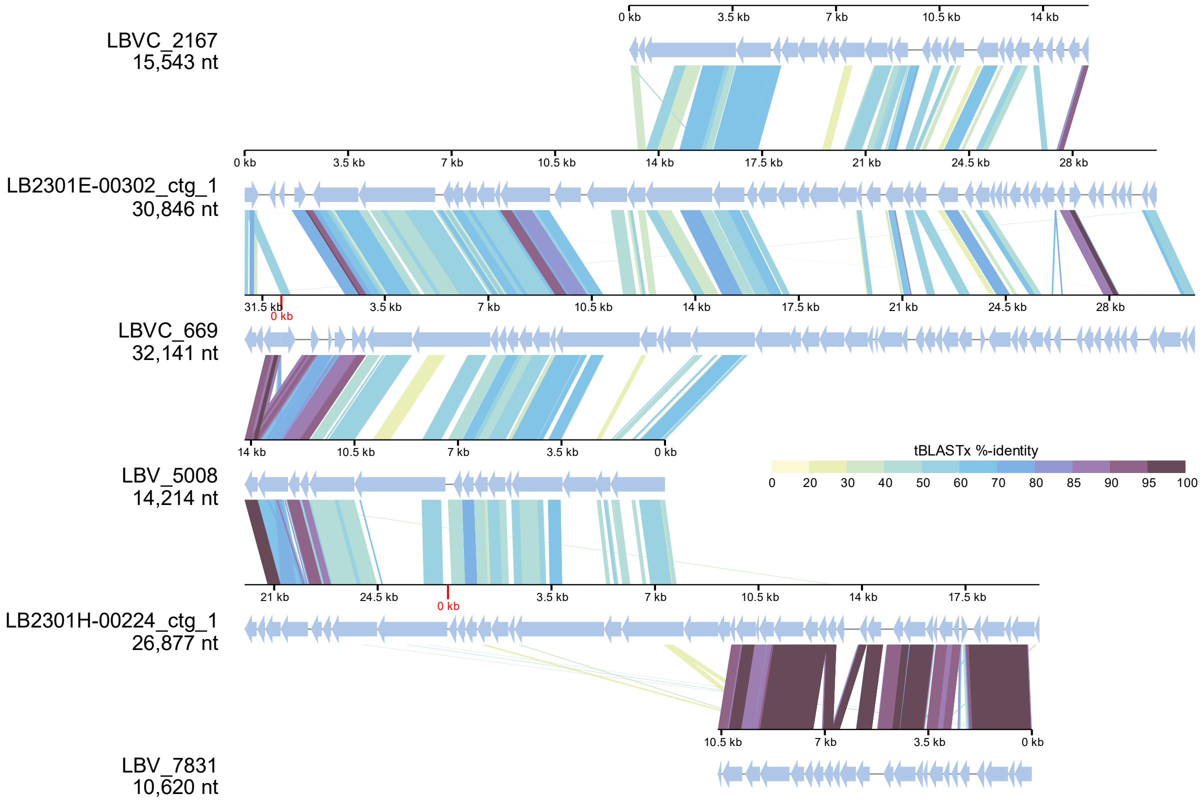

Fig. S10. tBLASTx alignment of the two circular CL500-11 viruses (LB2301H_00224_ctg_1 and LB2301E_00302_ctg_1) and their related reference viruses. Sequences may be inversed or circularly permuted using DiGAlign to show alignment more clearly.

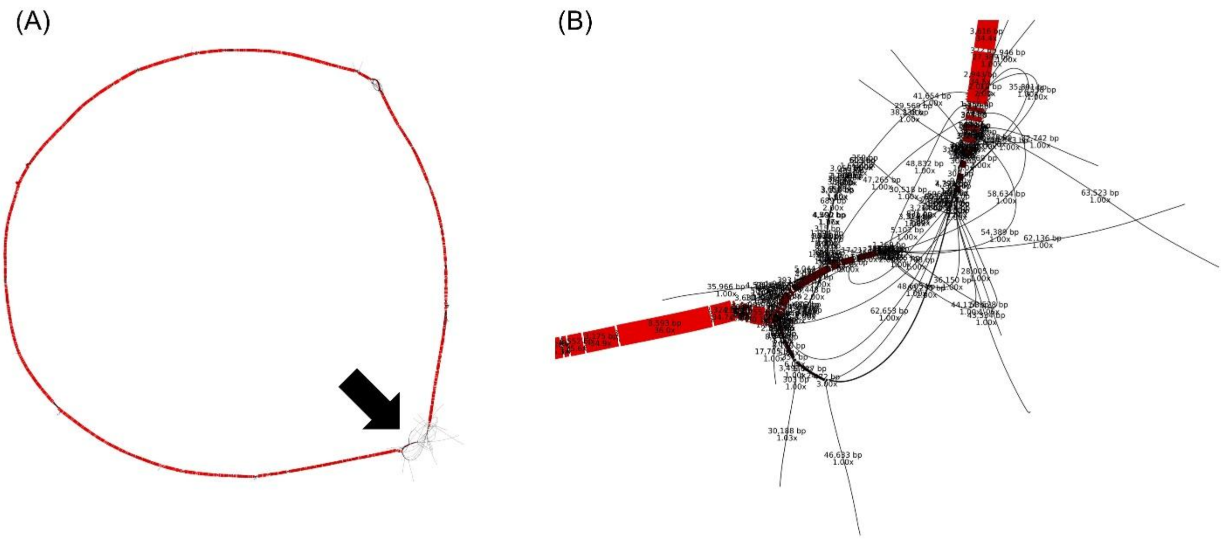

Fig. S11. Pangenome graph of the 39 SAGs with QS > 50 affiliated with *Fonsibacter ubiquis* collected from the summer epilimnion. Graph was generated by SuperPang using the default parameters and visualized by Bandage; genomic segments sharing > 95% similarity are shown as non-branching paths. Thus, inconsistent sequences between SAGs with < 95% sequence similarity are shown as branches in the graph. The width of a path indicates its coverage (average prevalence of its constituent kmers in input genomes). (A) Structure of the pangenome graph. Arrow indicates hypervariable region 2 (HVR2). (B) Enlarged HVR2 in the pangenome graph. Path labels indicate length and coverage.

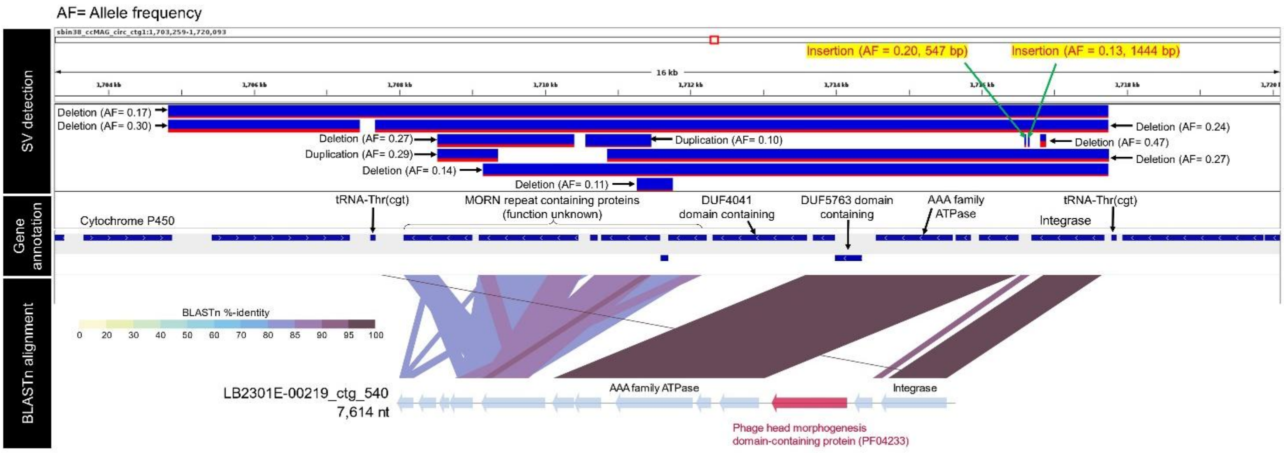

Fig. S12. The contig LB2301E-00219_ctg_540 aligned to a portion of a MAG of the host (CL500-11) shown with structural variants detected by metagenomic long-read mapping (Okazaki et al. 2022). The host MAG was assembled into a single circular contig; the segment from 1703 to 1720 kb is shown. The top layer shows the positions of detected structural variants (deletions, insertions, and duplications) with estimated allele frequencies. Insertions are shown with their estimated lengths. The middle layer shows gene annotations of the MAG in the original study (Okazaki et al. 2022). The bottom layer shows the BLASTn alignment between LB2301E-00219_ctg_540 and the MAG. Arrows indicate predicted genes. Red arrow indicates a phage head morphogenesis protein, which is absent in the MAG but present in the predicted insertion.

Captions for supplementary tables:

Table S1. Statistics of 1657 assemblies with a > 1-kb contig generated from individual gel beads. In total, 862 qualified (QS > 30) single-cell amplified genomes (SAGs) were obtained and used in downstream analyses.

Table S2. Statistics of 176 viral contigs assembled from 85 SAGs and detected as dsDNA phages by at least two of geNomad, VIBRANT, and VirSorter2 (see main text for detection criteria for each tool).

Table S3. Statistics of co-detected viral contigs. Two to nine viral contigs were co-detected in each of 55 SAGs, as indicated by different colors in the first and second columns of the table.
